# Effects of different concentrations of exogenous methyl jasmonate on tuber metabolism and rhizosphere bacterial community dynamics in *Polygonatum cyrtonema* Hua

**DOI:** 10.3389/fpls.2026.1950448

**Published:** 2026-10-28

**Authors:** Mingze Liu, Lingling Yan, Jiuxin Lai, Xiurong Xu, Shiming Cheng

**Affiliations:** 1 State Key Laboratory for Development and Utilization of Forest Food Resources, Zhejiang Agriculture and Forestry University, Hangzhou, China; 2 Zhejiang Academy of Forestry, Hangzhou, China; 3 Yongkang Forestry Technology and Seedling Extension Station, Yongkang, China

**Keywords:** methyl jasmonate, plant-microbe association, *Polygonatum cyrtonema* Hua, rhizosphere microbiome, secondary metabolism

## Abstract

*Polygonatum cyrtonema Hua* is a traditional medicinal and edible plant valued for its tubers, which are rich in bioactive components such as polysaccharides, flavonoids, and saponins. The phytohormone methyl jasmonate (MeJA) regulates plant secondary metabolism and stress responses, promoting the accumulation of medicinal compounds in *P. cyrtonema.* However, the temporal dynamics of the rhizosphere microbiome and its association with the tuber metabolism under different MeJA concentrations remain poorly understood. In this study, three-year-old *P. cyrtonema* plants were treated with rhizosphere sprays of 200 μmol L^−1^ and 400 μmol L^−1^ MeJA. Plants were sampled at 7, 14, 21, and 28 days post-treatment to evaluate tuber physiology and metabolite profiles, and metagenomic sequencing was used to characterize the structure and function of the rhizosphere bacterial community. Application of 200 μmol L^−1^ MeJA significantly enhanced tuber peroxidase (POD) activity, which peaked at 2.29 times that of the control on day 14 (*P* < 0.05), and increased total flavonoid and saponin contents by 22.58% and 13.85%, respectively (*P* < 0.05). In contrast, 400 μmol L^−1^ MeJA mainly inhibited antioxidant enzyme activities and delayed the induction of secondary metabolism. Metagenomic analysis revealed that the two MeJA concentrations shared the same dominant bacterial phyla but showed concentration-dependent differences in relative abundance dynamics, alpha diversity trajectories, and temporal patterns of community structure. The 200 μmol L^−1^ MeJA group exhibited a directional temporal shift (PERMANOVA R² = 0.79, P = 0.003) and continuously rising alpha diversity, whereas the 400 μmol L^−1^ treatment showed nonspecific stress fluctuations, with reduced alpha diversity, a weaker temporal gradient in community structure, and increased community homogenization. At the functional level, the 200 μmol L^−1^ MeJA group had higher relative abundances of microbial genes associated with nitrogen, carbon, and secondary metabolic pathways. In contrast, the 400 μmol L^−1^ MeJA group showed disrupted coordination among functional genes and persistently higher abundances of genes involved in antioxidant pathways. Correlation analysis identified associations between specific phyla and host traits: Nitrospirota was positively correlated with POD activity (r = 0.48, *P* < 0.05); Methylomirabilota was positively correlated with total flavonoid and saponin contents (r ≥ 0.41, *P* < 0.05); and Verrucomicrobiota was negatively correlated with these components (r < −0.55, *P* < 0.01). These findings suggest that 200 μmol L^−1^ MeJA promotes the accumulation of bioactive metabolites in *P. cyrtonema* through two proposed ways: the direct activation of host metabolism and the indirect association with shifts in potentially beneficial rhizosphere microbial taxa. This study provides a practical foundation for selective MeJA concentrations in medicinal plant cultivation.

## 1 Introduction

*Polygonatum cyrtonema* Hua is a perennial herbaceous plant in the genus *Polygonatum* (Asparagaceae). Its dried tubers are officially documented in the Chinese Pharmacopoeia (Chinese Pharmacopoeia Commission, 2020) and are traditionally used to nourish yin, moisten the lungs, invigorate the spleen, and replenish qi. Studies indicate that bioactive constituents of *P. cyrtonema* tubers, such as polysaccharides, flavonoids, and saponins, exert multiple pharmacological activities, including antioxidant, anti-inflammatory, and immunomodulatory effects (Huang et al., 2025). As market demand for high-quality *P. cyrtonema* continues to grow, developing eco-friendly cultivation techniques to improve its medicinal quality has emerged as a critical priority.

The rhizosphere acts as a key microenvironment for Plant-microbe association, shaping plant physiology and secondary metabolism through nutrient cycling, microbial hormone production, and induced systemic resistance (Berendsen et al., 2012). Recent studies indicate that the quality formation of medicinal plants is closely related to the composition and function of the rhizosphere microbiome (Chen et al., 2021). However, the specific dynamics of the *P. cyrtonema* rhizosphere microbiome and its functional association with host metabolism remain poorly understood.

Methyl jasmonate (MeJA) is a ubiquitous endogenous plant signaling molecule that mediates plant responses to biotic and abiotic stresses (Wasternack and Hause, 2013). Exogenous application of MeJA has been shown to enhance secondary metabolite accumulation in various medicinal plants by activating phenylpropanoid and terpenoid biosynthetic pathways (Zhou et al., 2017). Furthermore, previous studies have shown that MeJA can indirectly regulate the structure and temporal dynamics of rhizosphere bacterial communities by altering root exudate composition (Carvalhais et al., 2015). To date, most studies have investigated the direct effects of MeJA on host metabolism and its indirect effects on the microbiome as independent. Consequently, the complex associations among MeJA application, host metabolic responses, and rhizosphere dynamics remain largely unexplored. To bridge this gap, this study addresses the following objectives: (1) characterizing the temporal dynamics of rhizosphere bacterial communities under two MeJA concentrations; (2) determining the responses of key microbial taxa and functional pathways to MeJA elicitation; and (3) finding the correlations between these microbiomic shifts and the accumulation of host bioactive metabolites.

In this study, greenhouse experiments were conducted to investigate the dynamic effects of two representative MeJA concentrations (200 μmol L^−1^ and 400 μmol L^−1^, representing mild and stronger supraoptimal MeJA treatments) on the physiological metabolism of *P. cyrtonema* tubers and the structure and function of rhizosphere bacterial communities, and to screen key microbial taxa associated with host medicinal quality. This study aimes to elucidate the potential concentration-dependent associations through which MeJA may be linked to host phytochemistry and to provide a potential approach for cultivating high-quality medicinal *P. cyrtonema*.

## 2 Materials and methods

### 2.1 Plant materials and experimental design

The experiment was conducted in a greenhouse at the Zhejiang Academy of Forestry (30°15′ N, 120°07′ E). The greenhouse environmental parameters were as follows: average temperature, 28.3 °C; relative humidity, 68.8%; daytime light intensity, 350 μmol·m^−2^·s^−1^; soil temperature, 27–37 °C; soil water content, maintained at 70% of field capacity by daily irrigation; and CO_2_ concentration, 584–604 ppm. A total of 100 three-year-old *P. cyrtonema* Hua plants obtained from a previous variety breeding trial were cultivated in the greenhouse. The plants were propagated from tubers sourced from Yunhe, Zhejiang Province, and grown in sterilized garden soil in root-controlling pots (30 cm in diameter × 25 cm in height). The potting soil consisted of vermiculite and horticultural soil (PINGSTRUP SPHAGNUM, Denmark) mixed at a 1:2 volume ratio. Before cultivation, the soil was wrapped in gauze and sterilized by autoclaving at 121 °C for 60 min in a single cycle. After sterilization, the substrate was cooled to room temperature and used for potting and planting. The substrate pH was 5.82. No pesticides or chemical reagents were applied. Plants were irrigated daily to maintain soil water content at 70% of field capacity; no drought treatment was applied before sampling.

In this experiment, thirty-six *P. cyrtonema* plants were randomly selected and divided into three groups, with 12 plants in each group: (1) control group (CK), sprayed with an equal volume of distilled water; (2) low-concentration MeJA treatment group (J1), sprayed with 200 μmol L^−1^ MeJA; (3) high-concentration MeJA treatment group (J2), sprayed with 400 μmol L^−1^ MeJA.

The MeJA stock solution was prepared using anhydrous ethanol as a solubilizer and then diluted to the target concentrations with distilled water before spraying. In both treatment groups, the final anhydrous ethanol concentration was identical and below 0.1% (v/v). Because the final solvent concentration was identical in the two MeJA groups, differences in solvent concentration could not confound comparisons between them. Before treatment, plants were arranged in a completely randomized design to promote homogeneity of the rhizosphere microbial background among groups.

### 2.2 Sample collection

One control group and two treatment groups were established: control (0 μmol L^−1^ MeJA, CK), 200 μmol L^−1^ MeJA (J1), and 400 μmol L^−1^ MeJA (J2). At 7, 14, 21, and 28 days after MeJA treatment, three independent plants per group were harvested at each time point, and each plant was treated as one biological replicate. A total of 36 tuber samples were used for physiological and enzyme activity assays. Rhizosphere soil and tuber samples were collected at the same time. Rhizosphere soil samples were collected only from the J1 and J2 treatment groups, yielding 12 samples per treatment and 24 samples in total. Soil samples were immediately frozen in liquid nitrogen after collection and stored at −80 °C. The rhizosphere soil samples were reserved for metagenomic sequencing analysis.

### 2.3 Determination of physiological indices

Tuber water content (WC) was determined using the drying method and expressed on a fresh weight basis. The fresh weight (FW) of fresh tuber samples was measured first. After enzyme inactivation at 110 °C for 30 min, samples were dried to a constant weight at 55 °C, and the dry weight (DW) was recorded. The WC was calculated following:


WC (%)=[(FW − DW)/FW]×100%

Assay kits (superoxide dismutase, SOD: Cat. No. BC0170; peroxidase, POD: Cat. No. BC0090; Solarbio Science & Technology Co., Ltd., Beijing, China) were used in accordance with the instructions. 0.1 g of frozen tuber tissue was homogenized in 1 mL of pre-cooled extraction buffer (consisting of 50 mM sodium phosphate buffer, pH 7.8, 1 mM EDTA, and 1% (w/v) polyvinylpyrrolidone) in an ice bath and centrifuged at 8000 × g for 10 min at 4 °C. The supernatant was collected for enzyme activity assays. SOD activity was determined through the xanthine oxidase method, and POD activity was measured using the guaiacol oxidation method. One unit of POD activity was defined as the amount of enzyme required to increase the absorbance at 470 nm by 0.01 per minute.

### 2.4 Determination of medicinal components

The total polysaccharide content was determined by water extraction and alcohol precipitation combined with the phenol-sulfuric acid method, using a kit (Cat. No. DTP-2022-01; Suzhou Comin Biotechnology Co., Ltd., Suzhou, China). 0.05 g of dried tuber powder was mixed with 1 mL of distilled water and extracted in a 100 °C water bath for 2 h. After cooling, the mixture was centrifuged at 10, 000×g for 10 min. Then 0.2 mL of the supernatant was added to 0.8 mL of anhydrous ethanol and left to stand at 4 °C overnight. Following centrifugation, the resulting precipitate was dissolved in 1 mL of distilled water. 400 μL of the test solution was collected, mixed with 200 μL of phenol solution and 1 mL of concentrated sulfuric acid, and incubated in a 90 °C water bath for 20 min. The absorbance was measured at a wavelength of 490 nm, with glucose used as the standard substance.

The total flavonoid content was determined through the aluminum nitrate colorimetric method using a kit (Cat. No. DTF-2022-02; Suzhou Geruisi Biotechnology Co., Ltd., Suzhou, China). 0.03 g of dried tuber powder was added to 1.5 mL of 60% ethanol, followed by extraction with shaking at 60 °C for 2 h. After centrifugation at 12, 000 × g for 10 min, the absorbance of the resulting supernatant was measured at 510 nm.

The total saponin content was determined by the vanillin-perchloric acid colorimetric method with a kit (Cat. No. DTS-2022-03; Shanghai Yuanye Bio-Technology Co., Ltd., Shanghai, China). 0.1 g of dried powder was mixed with 10 mL of methanol and extracted at 60 °C for 2 h, followed by centrifugation at 8000×g for 10 min. The supernatant was evaporated to dryness. The residue was dissolved in methanol, mixed with vanillin-perchloric acid reagent, and heated at 60 °C for 15 min. The absorbance was measured at 540 nm, with ginsenoside used as the standard substance.

### 2.5 Rhizosphere soil metagenomic sequencing

Quality control metrics of the sequencing data were as follows: the Q20 ratio exceeded 97% and the Q30 ratio exceeded 92% across all samples, with an average GC content of 58.7%. A total of 1, 705, 068, 562 clean reads were obtained after quality control, with an average of 71, 044, 523 reads per sample. Quality filtering was performed using Trimmomatic v0.39 with a sliding window of 4 bp and an average quality threshold of Q20; reads with a length greater than 50 bp were retained for downstream analysis.

Total genomic DNA was extracted from 0.5 g of rhizosphere soil using the E.Z.N.A.^®^ Soil DNA Kit (Omega Bio-tek, USA). The concentration and purity of the extracted DNA were determined with a NanoDrop 2000 spectrophotometer, and DNA integrity was verified by 1% agarose gel electrophoresis.

Metagenomic sequencing libraries were constructed using the NEBNext^®^ Ultra™ DNA Library Prep Kit (NEB, USA), and paired-end 150 bp sequencing was performed on the Illumina NovaSeq 6000 platform. Raw reads were quality-filtered using Trimmomatic v0.39 to remove adapter sequences, low-quality reads (Q < 20), and reads containing ambiguous bases (Bolger et al., 2014).

Taxonomic annotation was conducted using Sylph v1.0: bacteria and archaea were aligned against the Genome Taxonomy Database (GTDB, release r214, accessed 15 March 2025), while fungi were aligned against the NCBI Reference Sequence Database (RefSeq, release 213, accessed 15 March 2025), with annotation thresholds set at ≥95% sequence identity and ≥80% coverage. Species abundance was normalized using the reads per kilobase per million mapped reads (RPKM) method to eliminate the effects of sequencing depth and gene length on abundance estimation (Conesa et al., 2016; Parks et al., 2018).

Functional annotation was performed using HMMER v3.3.2 by aligning sequences against the Kyoto Encyclopedia of Genes and Genomes (KEGG, release 104, accessed 20 March 2025) and the Carbohydrate-Active Enzymes database (CAZy, release 11, accessed 22 March 2025), respectively, with filtering thresholds of e-value < 1–^10^ and ≥30% sequence alignment coverage (Eddy, 2011). The abundance of functional genes was normalized using the TPM (Transcripts Per Million) method to ensure comparable functional abundance across samples.

### 2.6 Statistical analysis

Physiological and biochemical data were analyzed using SPSS 26.0 software. Prior to statistical analysis, all physiological and biochemical datasets were tested for normality by the Shapiro–Wilk method and for homogeneity of variance by Levene’s test. One-way analysis of variance (ANOVA) followed by Duncan’s multiple range test was applied to examine differences among different treatments at the same time when both assumptions were satisfied; for datasets that did not meet the assumptions, appropriate data transformation or the non-parametric Kruskal–Wallis H test was used instead, with the significance level set at *P* < 0.05. Data are expressed as mean ± standard deviation (SD). Microbiome data analysis was performed using R v4.2.1 with the vegan v2.6-4, phyloseq v1.42.0, and ggplot2 v3.4.0 packages (McMurdie and Holmes, 2013; Wickham, 2016; Oksanen et al., 2022). The Shannon, Simpson, Chao1, and ACE indices were calculated to evaluate alpha diversity, and intergroup differences were tested using the Wilcoxon rank-sum test, with *P* < 0.05 considered statistically significant.

Principal coordinate analysis (PCoA) and non-metric multidimensional scaling (NMDS) based on the Bray–Curtis distance matrix were conducted to characterize beta diversity. Permutational multivariate analysis of variance (PERMANOVA) was applied to test the significance of differences in community structure, with 9999 permutations. Analysis of similarities (ANOSIM) was performed to verify whether intergroup community differences were significantly greater than intra-group differences, with 9999 permutations.

Spearman’s rank correlation analysis was used to explore the associations between phylum-level microorganisms and host traits, while Pearson correlation analysis was applied to investigate the correlations between core taxa at the genus level and host traits. The correlation heatmap was generated based on the abundance matrix normalized by Z-score.

All correlation analyses were subjected to false discovery rate (FDR) correction for multiple testing using the Benjamini–Hochberg method, and an adjusted P value < 0.05 was regarded as a significant correlation.

## 3 Results

### 3.1 Effects of MeJA on physiological traits of tubers

#### 3.1.1 Tuber water content

The WC of tubers in the control group remained stable at approximately 81% throughout the 28-day sampling period. However, the WC in the J1 group was significantly lower than that in the control group at all time points (*P* < 0.05), reaching the minimum value of 66.75% on day 14. The J2 group resulted in an intermediate WC between the control and J1 groups, with the widest fluctuation range (67.38%–84.58%), peaking on day 21 (**Figure 1a**). Overall, MeJA treatment reduced the tuber WC of *P. cyrtonema*, and the inhibitory effect was more pronounced at the concentration of 200 μmol L^−1^.

**Figure 1:**
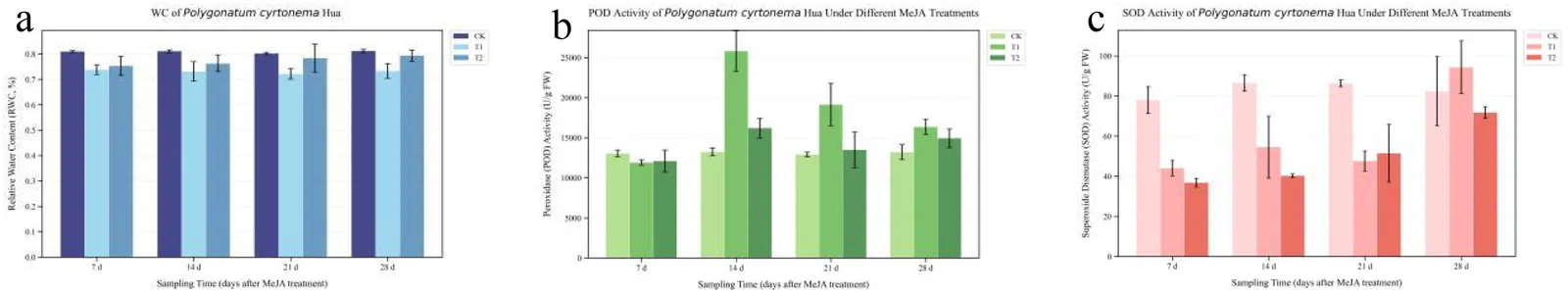
Bar chart of physiological changes in tubers after MeJA treatment. **(a)** WC. **(b)** POD activity. **(c)** SOD activity.

#### 3.1.2 Antioxidant enzyme activities

The results showed that POD activity in the control group remained stable at approximately 13, 000 U/g. POD activity in the J1 group peaked at 30, 172.59 U/g on day 14, which was 2.29 times that of the control group at the same time point (*P* < 0.05). This significant elevation was maintained until day 21, and then declined to a level showing no significant difference from the control group on day 28 (**Figure 1b**).In the J2 group, POD activity was significantly higher than that in the control group only on day 14 (*P* < 0.05), and no significant difference was detected at other time points.

For SOD activity, the control group showed a gradual upward trend, increasing from 77.90 U/g on day 7 to 82.32 U/g on day 28. The SOD activity in the J1 group showed initial decrease followed by late increase; it was significantly lower than that in the control group on days 7 and 14 (*P* < 0.05), and rose to 94.30 U/g on day 28, slightly above the control level (**Figure 1c**). The SOD activity in the J2 group was significantly lower than that in the control group throughout the entire sampling period (*P* < 0.05).

### 3.2 Effects of MeJA on medicinal components in tubers

#### 3.2.1 Total polysaccharide content

The results showed that the total polysaccharide content in the control group remained stable at approximately 126 mg/g. In the J1 group, the total polysaccharide content decreased significantly to 121.77 mg/g on day 7, recovered to match the control group on day 14, but was significantly lower than the control group on days 21 and 28.For the J2 group, the total polysaccharide content showed no significant difference from the control group in the first 14 days, then decreased continuously over time, reaching the minimum value of 121.75 mg/g on day 28, which was significantly lower than both the control group and the J1 group (*P* < 0.05) (**Figure 2a**). The total polysaccharide content under high-concentration MeJA treatment was significantly lower than that under the low-concentration group.

**Figure 2:**
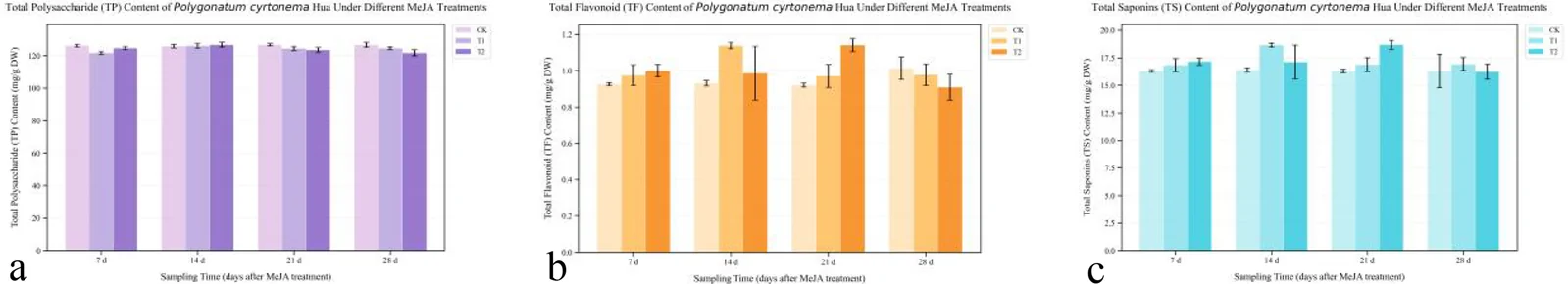
Bar chart of metabolite changes in tubers after MeJA treatment. **(a)** Total polysaccharides. **(b)** Total flavonoids. **(c)** Total saponins.

#### 3.2.2 Total flavonoid content

The total flavonoid content in the control group remained stable at approximately 0.93 mg/g, with a slight increase to 1.02 mg/g on day 28. In the J1 group, the total flavonoid content peaked at 1.14 mg/g on day 14, representing a 22.58% increase compared with the control group at the same time point, and then declined back to the control level. The peak of total flavonoid content in the J2 group appeared later than in the J1 group, reaching 1.14 mg/g on day 21, which was 23.91% higher than that of the control group; it then dropped significantly to 0.91 mg/g on day 28 (**Figure 2b**). Within the first 21 days of sampling, the total flavonoid content under both MeJA treatments was higher than that in the control group. The total flavonoid content in both groups had decreased to levels below that of the control group by day 28.

#### 3.2.3 Total saponin content

The total saponin content in the control group remained stable at approximately 16.3 mg/g. In the J1 group, the total saponin content peaked at 18.66 mg/g on day 14, which was 13.85% higher than that in the control group, and this significant elevation was maintained until day 21. The peak of total saponin content in the J2 group appeared on day 21 at 18.68 mg/g, representing a 14.67% increase compared with the control group; it then declined to a level not significantly different from that of the control group on day 28 (**Figure 2c**). Total saponin content was significantly increased under both concentrations of MeJA treatment.

### 3.3 Responses of rhizosphere bacterial community structure to MeJA

A total of 1, 705, 068, 562 clean reads (deposited in National Science Data Bank of China, with the data access link: https://www.scidb.cn/anonymous/MnVNSnJl) were retrieved from 24 rhizosphere soil samples in this study, with an average of 71, 044, 523 reads per sample. Taxonomic annotation identified three domains, 30 phyla, 47 classes, 89 orders, 153 families, 286 genera, and 412 species, and bacteria occupied over 99% of the total reads.

#### 3.3.1 Alpha diversity analysis and sequencing coverage

Alpha diversity reflects the species richness and evenness of the microbial community. Good’s coverage index of all samples in this study reached 1 (**Figures 3a, b**), confirming the reliability of the metagenomic sequencing results. Wilcoxon rank-sum analyses revealed that the rhizosphere bacterial alpha diversity exhibited significant differences between the two MeJA concentration treatment groups.

**Figure 3:**
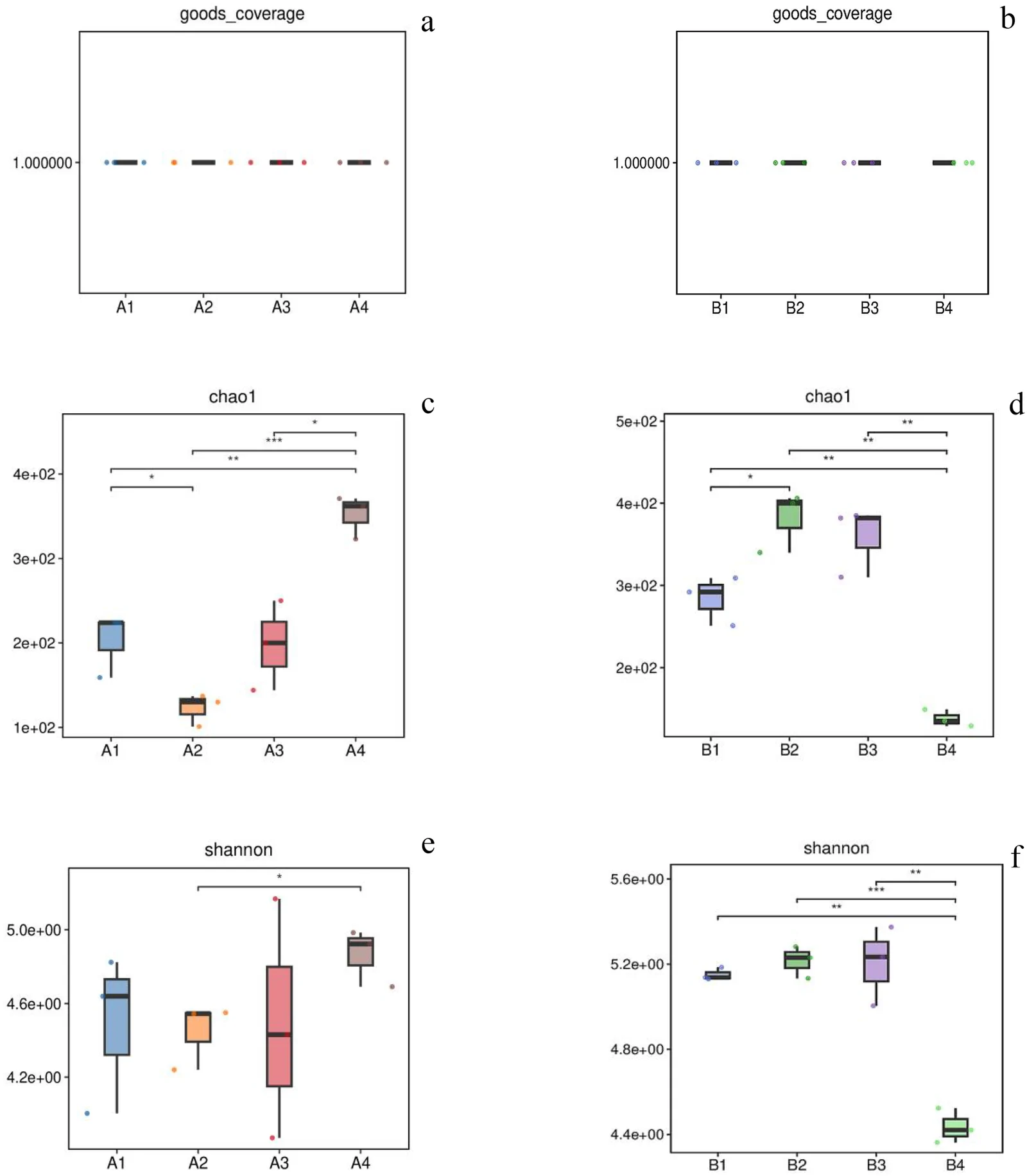
Alpha diversity analysis. **(a)** Good’s coverage of rhizosphere soil microorganisms under 200 μmol L^−1^ MeJA treatment. **(b)** Good’s coverage of rhizosphere soil microorganisms under 400 μmol L^−1^ MeJA treatment. **(c)** Chao1 index of rhizosphere soil microorganisms under 200 μmol L^−1^ MeJA treatment. **(d)** Chao1 index of rhizosphere soil microorganisms under 400 μmol L^−1^ MeJA treatment. **(e)** Shannon index of rhizosphere soil microorganisms under 200 μmol L^−1^ MeJA treatment. **(f)** Shannon index of rhizosphere soil microorganisms under 400 μmol L^−1^ MeJA treatment. The x-axis represents the experimental groups, with different groups distinguished by different colors; the y-axis represents the corresponding index values. A = J1, B = J2, A1, B1 = day7, A2, B2 = day14, A3, B3 = day21, A4, B4 = day28. A single asterisk (*) indicates a significant difference (*P* < 0.05). Double asterisks (**) indicate a highly significant difference (*P* < 0.01). Triple asterisks (***) indicate an extremely significant difference (*P* < 0.001).

Species richness was quantified using the Chao1 index. In group J1 (**Figure 3c**), species richness exhibited a continuous and significant upward trend over the sampling period. The median Chao1 index was 225 on day 7, and reached a peak of 355 on day 28, which was significantly higher than the values at all preceding time points (*P* < 0.05). In contrast, the species richness in group J2 (**Figure 3d**) remained at a relatively high level of 380–390 from day 14 to day 21, but dropped sharply to 150 on day 28, which was significantly lower than all previous sampling time points within the group (*P* < 0.01). Intergroup comparisons revealed that the Chao1 index of the J2 group was generally higher than that of the J1 group during the first 21 days after treatment. By day 28, however, the Chao1 index of the J1 group was significantly higher than that of the J2 group (*P* < 0.001). These results indicate that the temporal dynamics of rhizosphere bacterial species richness differed between groups: J1 showed a continuous enrichment trend over the sampling period, whereas J2 exhibited a significant decline in richness at the late stage of the experiment.

The Shannon index reflects the overall diversity of the community, and its variation trend was largely consistent with that of the Chao1 index. In group J1 (**Figure 3e**), the Shannon index reached 4.9 on day 28, which was significantly higher than the level on day 14 within the same group (pairwise Wilcoxon rank-sum test, *P* < 0.05). In group J2 (**Figure 3f**), the Shannon index decreased to 4.45 on day 28, which was significantly lower than all sampling time points within the group (all pairwise comparisons, *P* < 0.001), accompanied by a marked reduction in sample dispersion within the group.

Taken together, the alpha diversity of the 200 μmol L^−1^ MeJA group increased continuously throughout the sampling period. In contrast, the alpha diversity of the 400 μmol L^−1^ MeJA group showed an increase at the early stage, with both diversity and richness decreasing synchronously at the late stage.

#### 3.3.2 Beta diversity and temporal community dynamics

Beta diversity reflects the degree of dissimilarity in community structure among different samples. Phylum-level compositional analysis revealed consistent dominant bacterial phyla across all samples: Pseudomonadota (40.88%), Actinomycetota (19.00%), Acidobacteriota (11.94%), Gemmatimonadetes (11.01%) and Thaumarchaeota (7.32%), which collectively accounted for 89.15% of the total community abundance (**Figures 4e, f).**

**Figure 4:**
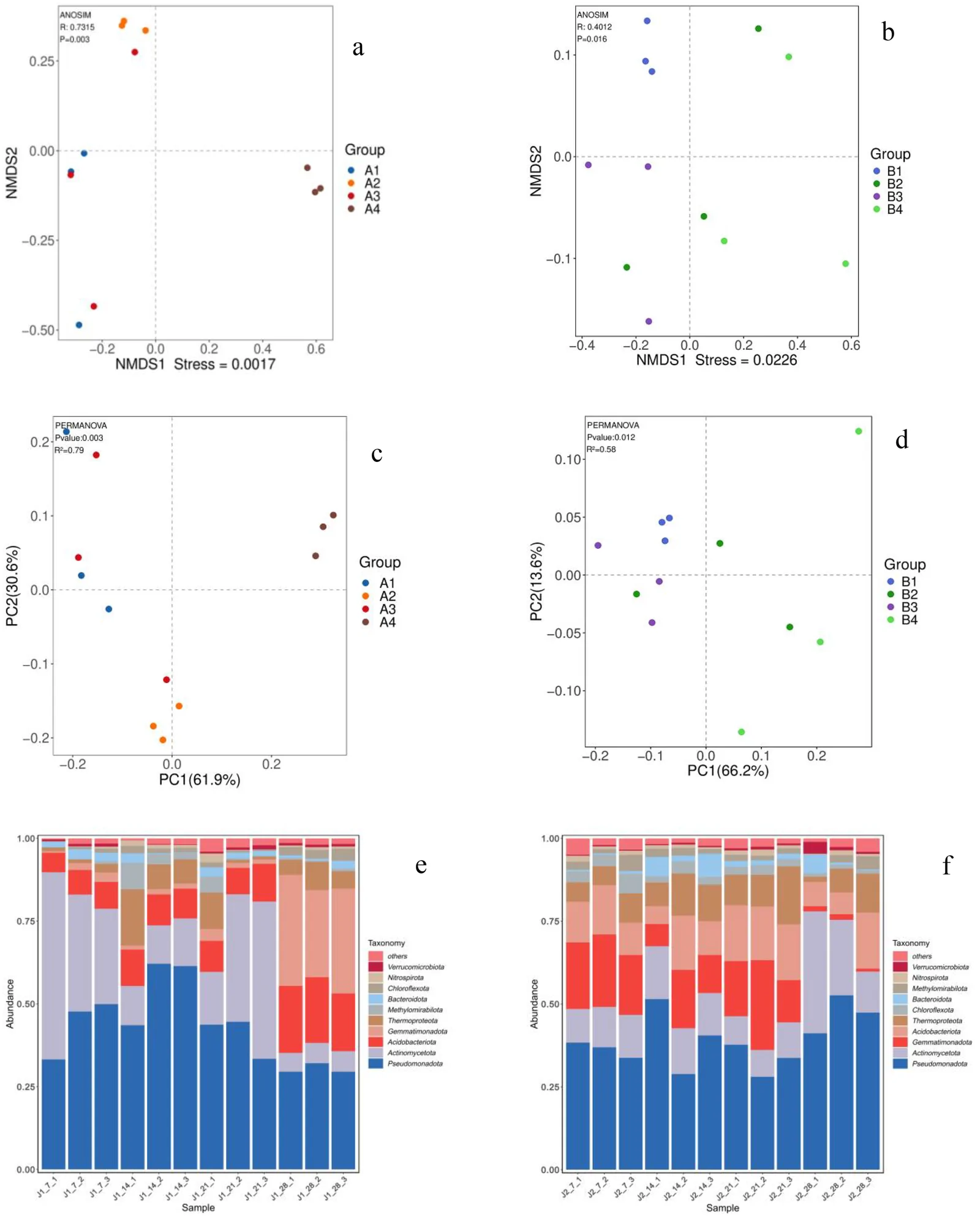
Beta diversity analysis. **(a)** NMDS ordination of rhizosphere soil microorganisms under 200 μmol L^−1^ MeJA treatment. **(b)** NMDS ordination of rhizosphere soil microorganisms under 400 μmol L^−1^ MeJA treatment. **(c)** PCoA ordination of rhizosphere soil microorganisms under 200 μmol L^−1^ MeJA treatment. **(d)** PCoA ordination of rhizosphere soil microorganisms under 400 μmol L^−1^ MeJA treatment. **(e)** Stacked bar chart of the top 10 most abundant bacterial phyla under 200 μmol L^−1^ MeJA treatment. **(f)** Stacked bar chart of the top 10 most abundant bacterial phyla under 400 μmol L^−1^ MeJA treatment. A and J1 correspond to the 200 μmol L^−1^ MeJA treatment, while B and J2 correspond to the 400 μmol L^−1^ MeJA treatment; 1, 2, 3 and 4 represent the four sampling time points respectively. A Stress value < 0.2 indicates reliable NMDS ordination results.

The taxonomic composition of dominant rhizosphere prokaryotic phyla was highly consistent across the two MeJA treatment groups, whereas the temporal dynamics of their relative abundances showed significant concentration-dependent differences. In the J1 group, the relative abundance of Pseudomonadota peaked on day 14, while that of Actinomycetota decreased significantly. In the J2 group, Pseudomonadota consistently ranked as the most dominant phylum, and the relative abundance of Actinomycetota increased gradually over time. Furthermore, the relative abundances of Nitrospirota, Methylomirabilota and Chloroflexi in both groups showed an increasing trend with sampling time. NMDS and PCoA analyses revealed significant differences in temporal community trajectories under different MeJA concentrations (**Figures 4a–d**). The NMDS Stress value was 0.0017, and the first two principal coordinates of PCoA jointly explained 92.5% of the total community variation.

In the J1 group, PERMANOVA (R2 = 0.79, P = 0.003) and ANOSIM (R = 0.7315, P = 0.003) indicated that treatment time was the main explanatory variable for community structural changes. In the J2 group, separate PERMANOVA and ANOSIM models were used, and the R2 values from the two groups were not formally compared. The proportion of community variation was 0.79 in J1 and 0.58 in J2, based on separate PERMANOVA models; these values were not formally compared. Day 21 appeared to be a key point for community structure alteration. In contrast, samples in the J2 group did not exhibit a distinct temporal trajectory, displaying greater within-group variance, particularly on days 14 and 21. Within the J2 group, days accounted for 58% of the community variation (PERMANOVA R² = 0.58, P = 0.012), and the ANOSIM R value was 0.4012 (P = 0.016); the corresponding value in J1 was 0.7315 (P = 0.003). These findings indicate that the community structure under 200 μmol L^−1^ MeJA group exhibits directional temporal shift. Conversely that in the 400 μmol L^−1^ MeJA group lack a clear temporal trajectory and exhibits an increased degree of homogenization at the late stage of the experiment.

### 3.4 Functional profile analysis of the rhizosphere microbiome

#### 3.4.1 KEGG level 2 functional annotation

KEGG Level 2 functional annotation revealed comparable core functional compositions between the two groups, with the relative abundance of “global and overview maps” maintaining a narrow range of 40.9%–41.8%. This indicates that the fundamental metabolic network of the rhizosphere microbiome remained stable (**Figure 5**).

**Figure 5:**
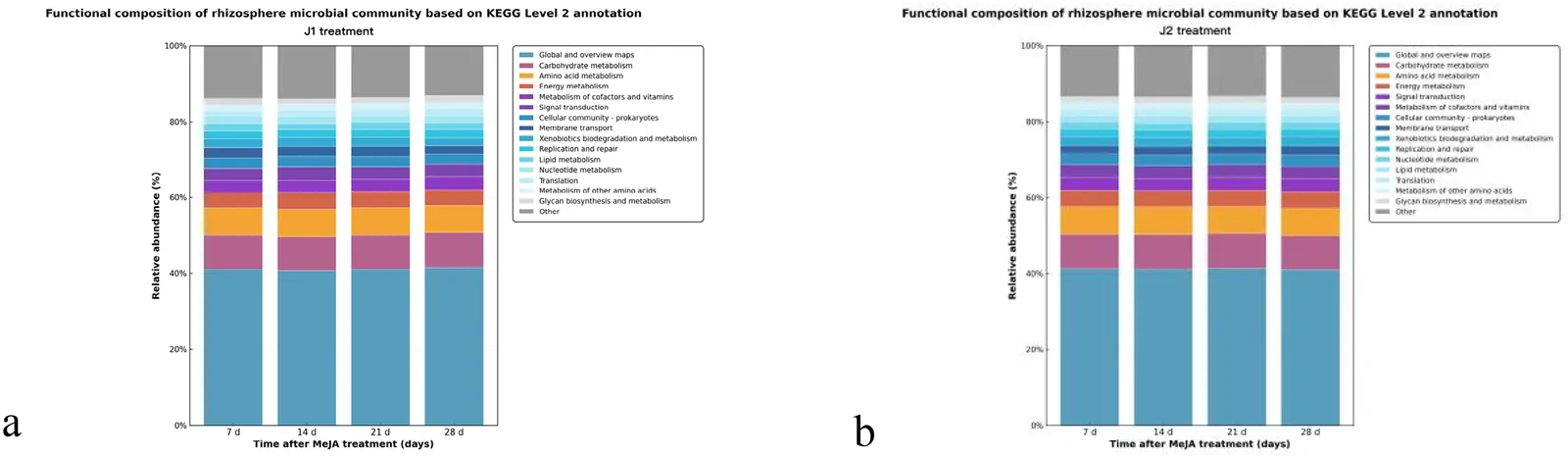
Stacked bar chart of KEGG Level 2 functional annotation. **(a)** J1 treatment. **(b)** J2 treatment.

However, the temporal dynamics of functional profiles exhibited remarkable concentration-dependent differences. In the J1 group (200 μmol L^−1^ MeJA), the relative abundance of genes annotated to the carbohydrate metabolism pathway peaked significantly on day 14 after treatment, with no significant variations at other time points. In the J2 group, the relative abundances of genes annotated to energy and xenobiotics biodegradation metabolism pathways increased over the obersvation period (**Figure 5b**).

#### 3.4.2 KEGG level 3 functional annotation

Annotation at KEGG Level 3 highlighted concentration-dependent in specific metabolic pathway (**Figure 6**). While the temporal dynamics of the nitrogen metabolism diverged significantly between the groups, the J1 group (200 μmol L^−1^ MeJA) displayed a specific response in carbohydrate metabolism, where related gene abundances were significantly higher on day 14 and remained relatively consistent at other time points. Compared with the day 7 within the group, the relative abundance on day 14 was significantly increased by 18.2% (*P* < 0.05), and the variation trend was consistent with the abundance dynamics of Nitrospirota. In the J2 group (400 μmol L^−1^ MeJA), genes annotated to the nitrogen metabolism pathway maintained a consistently high relative abundance throughout the entire experimental period from day 7 to day 28, with no significant temporal fluctuation. Overall, the mean relative abundance of this pathway was significantly higher in the J2 group by 12.5% compared to the J1 group (*P* < 0.05) (**Figures 6a, b**).

**Figure 6:**
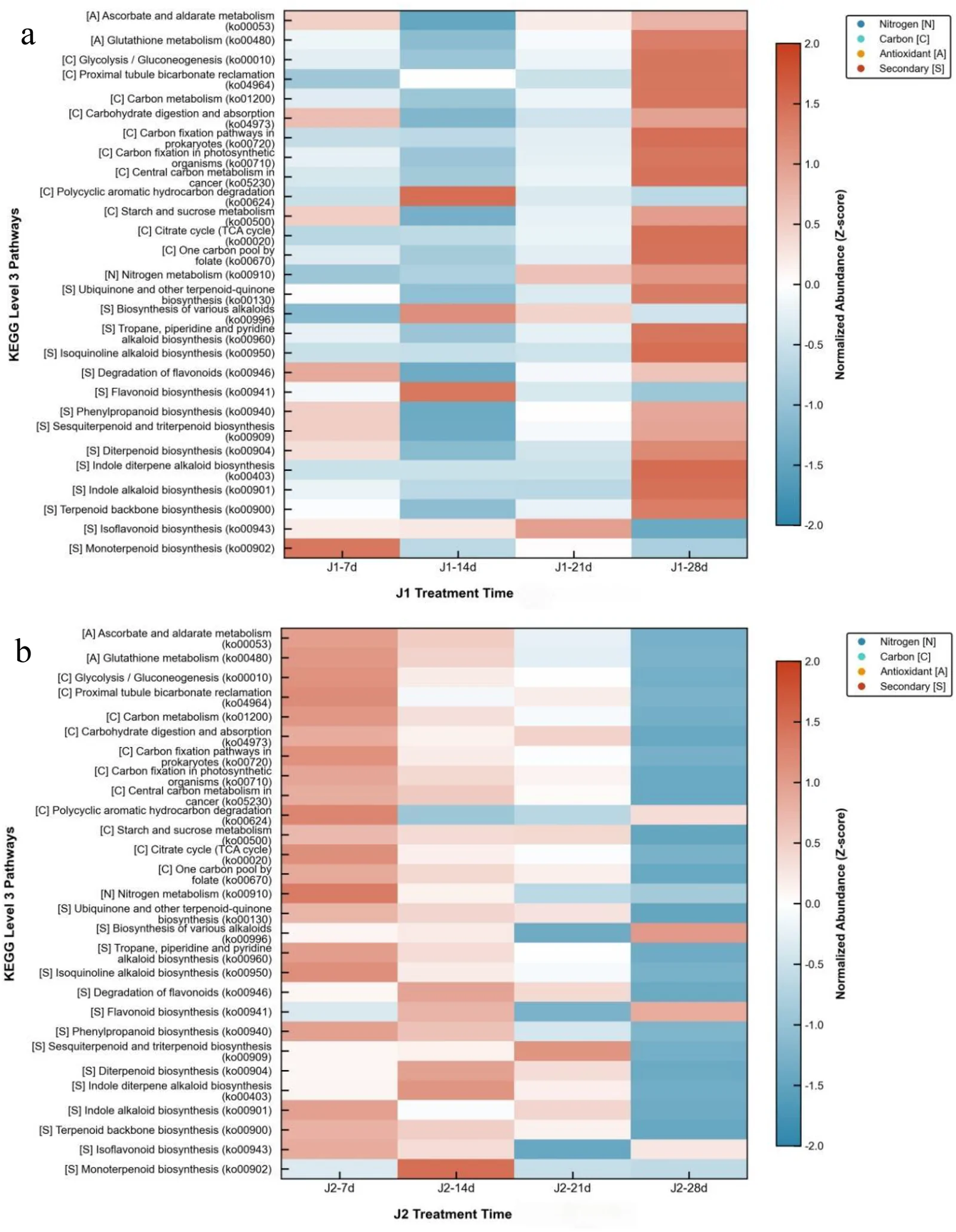
Heatmap of differentially enriched KEGG Level 3 functional pathways. **(a)** J1 treatment. **(b)** J2 treatment.

In the J1 group, genes related to glycolysis and the TCA cycle peaked on day 14 (showing significant increases of 22.4% and 19.7%, respectively; *P* < 0.05). Moreover, the relative abundance of genes annotated to the starch and sucrose metabolism pathway was positively correlated with the polysaccharide content in host tubers (r = 0.48, *P* < 0.05). In contrast, the J2 group exhibited no synchronied peaking among carbon metabolism pathways, the average relative abundance of the TCA cycle pathway was 9.3% lower compared to the J1 group (*P* < 0.05) (**Figures 6a, b**).

The glutathione metabolism and peroxisome pathways remained stable in the J1 group, with a fluctuation amplitude of less than 3.5%. By contrast, the abundances of these two pathways in the J2 group increased continuously over time, rising by 27.6% and 21.3% respectively on day 28 relative to day 7 (*P* < 0.05).

Within the J1 group, genes related to phenylpropanoid and terpenoid backbone biosynthesis showed higher relative abundances from day 14 to 21 (showing significant increases of 25.1% and 20.8%, respectively; *P* < 0.05). These trends paralleled the temporal accumulation peaks of flavonoids and saponins in the host. In contrast, the J2 group showed significantly lower abundances across these secondary metabolism pathways. Notably, the abundance of the “Flavonoid biosynthesis” pathway was 15.6% lower than that observed in the J1 group (*P* < 0.05).

#### 3.4.3 CAZy carbohydrate-active enzyme annotation

Annotation of carbohydrate-active enzymes (CAZymes) based on the CAZy database (e-value < 1e-10) revealed highly consistent functional distribution patterns of CAZyme genes between the two groups (**Figure 7**). Glycoside Hydrolases (GHs) and Glycosyltransferases (GTs) represented the most abundant categories; GH and GT had 61, 582 and 41, 427 non-redundant genes in J1, and 59, 936 and 40, 825 in J2, respectively. They were followed by Carbohydrate Esterases (CEs), Auxiliary Activities (AAs) and Carbohydrate-Binding Modules (CBMs). Polysaccharide Lyases (PLs) had the smallest number of genes, with 3, 400 and 3, 219 genes detected in the two groups, respectively.

**Figure 7:**
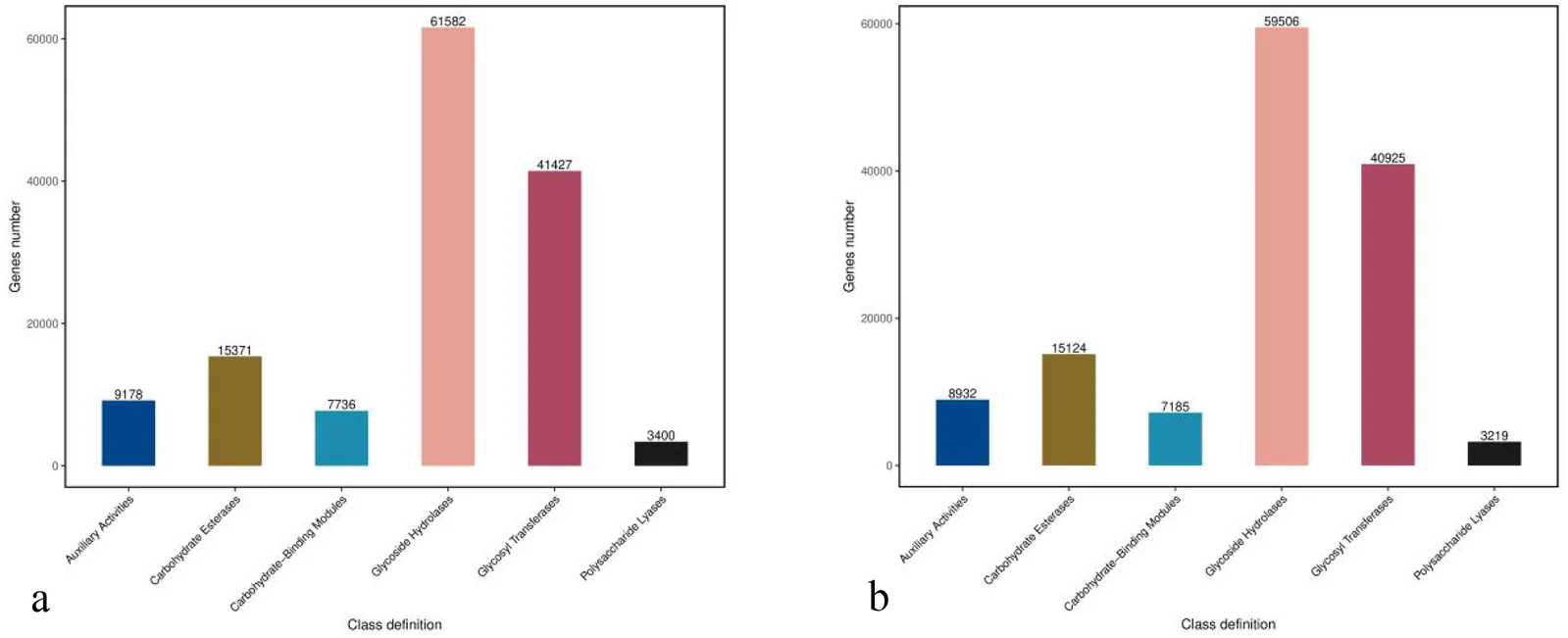
Bar chart of the number of genes annotated against the CAZy database. **(a)** J1 treatment. **(b)** J2 treatment.

Compared with the J1 group (**Figure 7a**), the gene numbers of all six CAZy functional categories in the J2 group (**Figure 7b**) exhibited a declining trend. CBMs showed the greatest reduction (7.1%), followed by PLs (5.3%), whereas GHs, AAs, CEs and GTs decreased by 3.4%, 2.7%, 1.6% and 1.2%, respectively.

These results indicate that the total number of carbohydrate-active enzyme genes in the high-concentration group is generally lower than that in the low-concentration group, which is consistent with the variation trend of carbon metabolism pathways.

### 3.5 Correlation between rhizosphere microbiome and host traits

#### 3.5.1 Associations between key phylum-level taxa and host traits

After FDR correction for multiple testing, Spearman rank correlation analysis identified three bacterial phyla significantly associated with host traits (**Figure 8**). Nitrospirota was significantly positively correlated with POD activity (Spearman r = 0.48, adjusted *P* < 0.05). Methylomirabilota showed significant positive correlations with both total flavonoid content (r = 0.41, adjusted *P* < 0.05) and total saponin content (r = 0.42, adjusted *P* < 0.05). Verrucomicrobiota exhibited significant negative correlations with total flavonoid content (r = −0.55, adjusted *P* < 0.01) and total saponin content (r = −0.57, adjusted *P* < 0.01).

**Figure 8:**
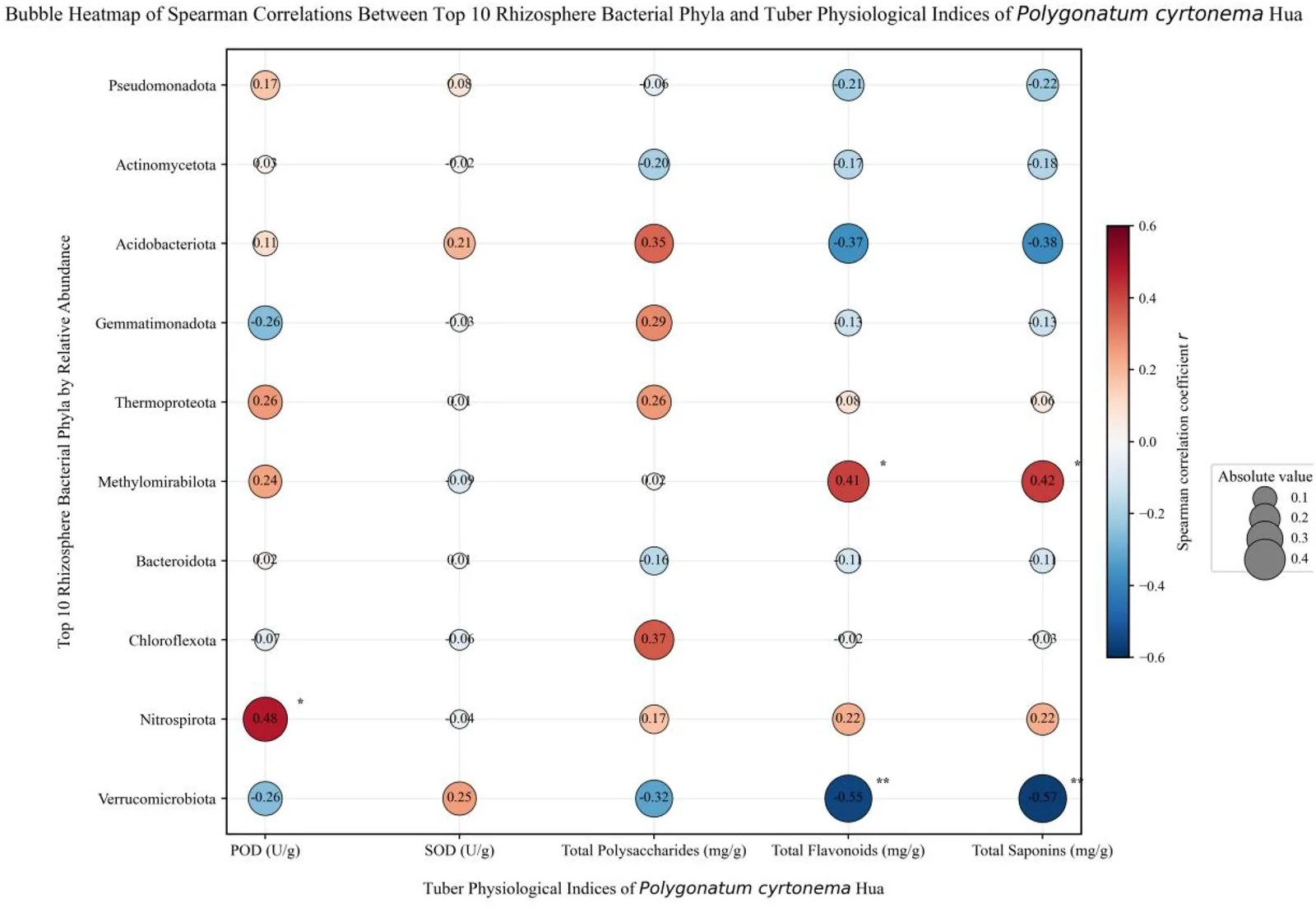
Bubble heatmap of Spearman correlations between the top 10 rhizosphere bacterial phyla and tuber physiological indices of *Polygonatum cyrtonema* Hua.

Additionally, Acidobacteriota and Chloroflexi tended to be positively correlated with total polysaccharide content, whereas Pseudomonadota and Actinomycetota showed no significant correlations with any of the measured indices (all adjusted *P* > 0.05).

#### 3.5.2 Identification of functional associations among dominant genera

To further validate the phylum-level associations, the top 30 genera ranked by relative abundance were selected for Pearson correlation analysis with five key host indices (**Figure 9**). On one hand, these donminant taxa (relative abundance > 90%) are presumed to be the primary drivers of rhizosphere microbial ecological processes. On the other hand, rare taxa (relative abundance < 0.1%) are affected by sequencing noise and exhibit insufficient statistical power.

**Figure 9:**
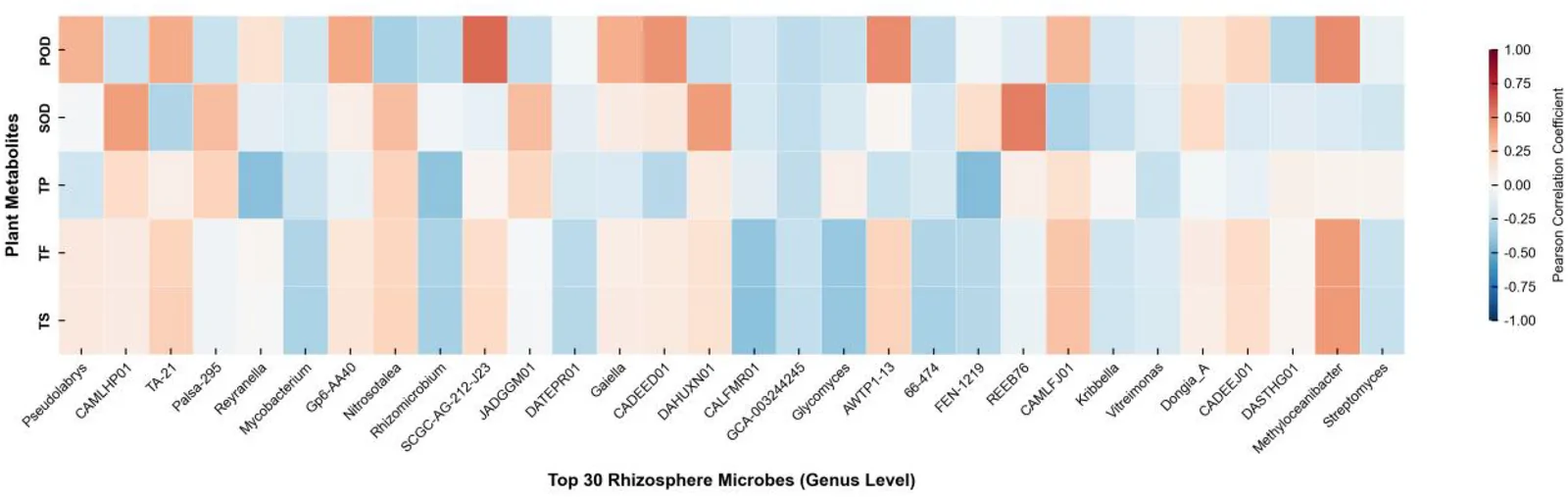
Associations between the top 30 dominant genera and host traits.

Accordingly, this study focused on dominant genera for further analysis. The results showed that after FDR correction, most microbial genera exhibited significant correlations only with specific host metabolic indices, presenting a high degree of functional specificity. No single genus was found to be significantly and strongly correlated with all five indices simultaneously. After FDR correction, four genera with significantly strong positive correlations with POD activity were identified (adjusted *P* < 0.05, Pearson r > 0.5), namely SCGC-AG-212-J23 (Actinomycetota, Acidimicrobiia), DAHUXN01 (Pseudomonadota, AlphaPseudomonadota), AWTP1-13 (Pseudomonadota, GammaPseudomonadota), and *Methyloceanibacter* (Pseudomonadota, AlphaPseudomonadota). In addition, seven genera showed significantly moderate positive correlations with POD activity (adjusted *P* < 0.05, 0.25 < r < 0.5). Only REEB76 (Actinomycetota, Actinomycetes) exhibited a significantly strong positive correlation with SOD activity (adjusted *P* < 0.05, r > 0.5), and another five genera showed significantly moderate positive correlations. *Reyranella* (Pseudomonadota, AlphaPseudomonadota), *Rhizomicrobium* (Pseudomonadota, AlphaPseudomonadota), and FEN-1219 (Acidobacteriota) were significantly and strongly negatively correlated with total polysaccharide content (adjusted *P* < 0.05, r < -0.5).

*Mycobacterium* (Actinomycetota, Actinomycetes), CALFMR01 (Actinomycetota, Actinomycetes), and GCA-003244245 (Actinomycetota, Actinomycetes) showed significantly strong negative correlations with both total flavonoids and saponin contents. In contrast, *Methyloceanibacter* (Pseudomonadota, AlphaPseudomonadota) was the only genus exhibiting a significantly strong positive correlation with total saponin accumulation. Based on the corrected association patterns, the 30 dominant genera could be divided into two distinct clusters: a positive correlation cluster (e.g., Nitrosotalea, DATEPR01, TA-21, etc.), which showed significant positive correlations with most host indices, and a negative correlation cluster (e.g., Rhizomicrobium, FEN-1219, CAMLFJ01, etc.) that exhibited significant negative correlations with host polysaccharide, flavonoid, and saponin contents.

## 4 Discussion

### 4.1 Concentration-dependent effects of MeJA on metabolism in *P*. *cyrtonema* Hua

As a classic elicitor of plant defense responses, exogenous MeJA exhibits a pronounced concentration-dependent effect. 200 μmol L^−1^ MeJA significantly activated POD activity in *P*. *cyrtonema* tubers and effectively elevated total flavonoids and total saponin contents on day 14. 400 μmol L^−1^ MeJA primarily suppressed antioxidant enzyme activity and delayed the induction of secondary metabolism. This is consistent with previous reports on medicinal plants, such as *Salvia miltiorrhiza* and *Panax ginseng* (Sohn et al., 2022), suggesting that moderate MeJA concentrations may enhance antioxidant capacity and promote secondary metabolism, whereas excessive concentrations are associated with oxidative stress and metabolic disorders (Sohn et al., 2022).

Interestingly, both MeJA treatments suppressed polysaccharide accumulation. This differential regulation may arise from the redistribution of carbon flux: in MeJA-triggered defense responses, plants prioritize carbon allocation for synthesizing defense-related secondary metabolites such as flavonoids and saponins, which in turn reduces polysaccharide synthesis dedicated to energy storage (Schwachtje and Baldwin, 2008). Furthermore, the MeJA-induced decline in tuber WC may indirectly restrict polysaccharide synthesis by altering cellular osmotic balance (Zhong et al., 2011).

### 4.2 Temporal dynamics and response patterns of the rhizosphere microbiome under different MeJA concentrations

The composition and dynamics of the rhizosphere microbiome serve as an integrated manifestation of plant–environment association. Comparative analysis of the two MeJA treatments showed that the taxonomic composition of dominant bacterial phyla was consistent across both concentrations, while the temporal dynamic trajectories of the bacterial community exhibited significant concentration-dependent differences. The 200 μmol L^−1^ group was associated with a directional temporal shift, with progressively increasing alpha diversity. In contrast, 400 μmol L^−1^ group showed nonspecific stress fluctuations, with reduced community stability and predictability. This divergence may be attributed to the differential regulatory effects of MeJA on tuber exudate profiles. This potential association between jasmonate signaling and rhizosphere microbial community assembly via altered root exudate composition has been reported in Arabidopsis systems with untreated wild-type controls (Carvalhais et al., 2015). At low concentrations, MeJA acts as a specific signaling molecule that induces plants to secrete targeted tuber exudates, associated with higher relative abundances of potentially beneficial microbial taxa. At high concentrations, MeJA elicits nonspecific stress responses in plants, which alters the overall composition of root exudates and is associated with disordered fluctuations in the microbial community. Additionally, oxidative stress induced by high-concentration MeJA may directly inhibit the growth of sensitive microbial taxa, further reducing the diversity and functional stability of the community.

This concentration-dependent divergence is consistent with the emerging view that MeJA acts as a mobile signal in the rhizosphere. Kulkarni et al. (2024) demonstrated that root-derived volatile MeJA rapidly triggers soil microbiome biofilm formation and alters community composition, extending the sphere of host influence beyond the root exudate zone. Our results extend this concept by showing that the direction and stability of rhizosphere bacterial temporal dynamics are strongly dependent on MeJA concentration.

### 4.3 Potential microbe-host association patterns associated with MeJA-induced quality improvement

This study identifies three key bacterial phyla significantly associated with the medicinal quality of *P. cyrtonema*, providing candidate mechanistic clues for how varying MeJA concentrations may modulate host metabolism in association with the rhizosphere microbiome (**Figure 10**). Exogenous MeJA was associated with concentration-dependent shifts in the rhizosphere microbiome of *P. cyrtonema*, and these microbiome shifts are associated with changes in host metabolism. The 200 μmolL^−1^ group was associated with directional temporal shifts and elevated diversity of rhizosphere bacteria, higher relative abundances of genes annotated to nitrogen, carbon, and secondary metabolic pathways, and higher relative abundances of potentially beneficial taxa such as Nitrospirota and Methylomirabilota. Conversely, the 400 μmolL^−1^group was associated with reduced alpha diversity, weaker temporal gradients in community structure, and metabolic changes, which coincided with delayed and unstable induction of host secondary metabolism.

**Figure 10:**
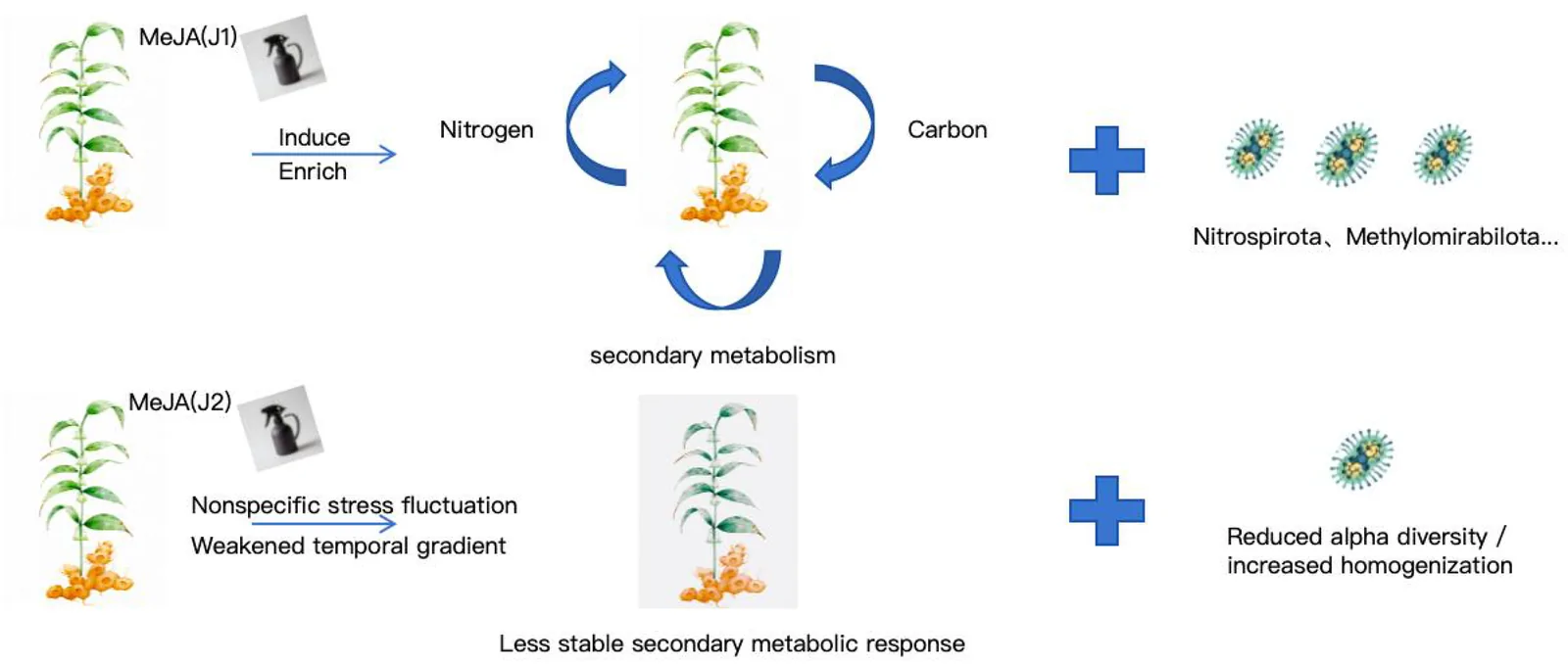
Schematic diagram of the potential dual association pattern of MeJA in regulating the quality of *P. cyrtonema* Hua.

Specific taxonomic association patterns are consistent with the hypothesis of microbiome-associated modulation. Nitrospirota, a well-documented taxon involved in soil nitrogen cycling (Daims et al., 2015), was positively correlated with host POD activity. This association may be linked to its role in increasing soil nitrogen availability, which could potentially support plant antioxidant capacity. Similarly, Methylomirabilota is a bacterial group capable of anaerobic methane oxidation that widely participates in soil carbon cycling. Its abundance in the present study was significantly positively correlated with both total flavonoids and total saponin contents after FDR correction. This association indicates that it may be involved in host secondary metabolism processes via participation in soil carbon cycling regulation or secretion of active signaling molecules (Guerrero-Cruz et al., 2021), but the causal relationship remains to be verified. Conversely, Verrucomicrobiota was negatively correlated with the contents of medicinal components. This negative association may be related to nutrient competition with plants or the production of metabolites that potentially suppress secondary metabolism, yet the underlying mechanism requires further investigation.

Functional annotation results are consistent with the above associative patterns. The 200 μmol L^−1^ group showed higher relative abundances of microbial genes involved in nitrogen metabolism, carbon metabolism, and secondary metabolic pathways. These shifts in functional gene abundance were associated with the accumulation of medicinal components in the host, potentially via providing abundant nutrients and precursors. Meanwhile, the 400 μmol L^−1^ group showed disrupted coordination of functional gene profiles and persistently higher relative abundances of genes involved in antioxidant pathways, which may indicate a stress-related functional signature. This stress state was associated with the less stable induction of host secondary metabolism. Furthermore, CAZy annotation revealed that high-concentration MeJA was associated with lower gene abundances of carbohydrate-active enzymes. This reduction may potentially impair the capacity of microorganisms to degrade soil organic matter, which could in turn be linked to restricted nutrient supply to the host.

Ultimately, this study proposes a two-pronged associative pattern for the concentration-dependent regulation of *P. cyrtonema* quality by MeJA. First, MeJA directly participates in the regulation of the host antioxidant system and secondary metabolic processes after being absorbed by roots. Second, 200 μmol L^−1^ MeJA was associated with directional temporal shifts in the rhizosphere microbial community, higher relative abundances of potentially beneficial taxa including Nitrospirota and Methylomirabilota, and enhanced soil nutrient cycling potential.

### 4.4 Limitations and future perspectives

We acknowledge several methodological limitations in the present study. While investigations of MeJA regulation in related medicinal plants typically employ comprehensive concentration gradients and untreated controls to establish dose–response relationships (Kandoudi and Németh-Zámboriné, 2022). Zhang et al. (2022) reported that an appropriate concentration of exogenous MeJA promoted nutrient uptake from the rhizosphere soil, alleviated soil acidification, enhanced soil enzyme activities. However, these effects were reversed as the MeJA concentration (over 100μmol L^−1^) continued to increase, 200 μmol L^−1^MeJA has a slight inhibitory effect on soil enzyme activity, while 500 μmol L^−1^ shows significant inhibition. We found that concentrations of 200 μmol L^−1^and 400 μmol L^−1^ have inhibitory effects on POD enzyme activity, and this effect gradually weakens over time. Interestingly, we found that 200 μmol L^−1^ and 400 μmol L^−1^ did have a promoting effect on the activity of SOD enzyme, and significant at the 200 μmol L^−1^. However, no study has provided an explanation from the perspective of the rhizosphere microbiome in *P. cyrtonema*. Our primary objective was to compare the relationship and attenuation between tuber physiological metabolism and rhizosphere microbial changes at different sampling time points following the application of two supraoptimal MeJA concentrations (200 and 400 μmol L^−1^). For future experiments, setting up a control group with a 0 μmol L^−1^ will be more scientific.

Another limitation is that the current design cannot fully separate direct host responses from microbiome-mediated effects, because MeJA applied to the tuber zone may simultaneously act on the host and the soil microbiota. Future work using axenic plant systems or split-root assays combined with reinoculation would help disentangle these pathways (Kafle et al., 2022). Because this study focused on bacterial communities, fungal and other microbial groups were not assessed, and the microbe–metabolite relationships remain correlational (Tedersoo et al., 2020; Li et al., 2024; Trivedi et al., 2020). Integrating multi-kingdom profiling, host transcriptomics, and functional validation would help clarify the mechanisms and causality.

Finally, the greenhouse pot experiment cannot fully represent field conditions. Multi-site and multi-year trials are therefore needed to verify the practical potential of MeJA application (Ramírez-López et al., 2025). Future research should focus on (1) integrating multi-omics approaches to decipher microbe–host association, (2) isolating and applying the beneficial taxa identified here, and (3) validating MeJA-based management under field conditions.

## 5 Conclusions

This study examined the effects of exogenous MeJA on tuber metabolism and the rhizosphere microbiome of *P*. *cyrtonema*. The results indicate that 200 μmol L^−1^ MeJA may enhance the medicinal components of *P. cyrtonema*, by stimulating POD activity and promoting flavonoid and saponin accumulation. The 400 μmol L^−1^ MeJA was associated with oxidative stress, leading to an unstable and delayed secondary metabolic response. These physiological shifts were paralleled by distinct rhizosphere microbiome dynamics, the 200 μmol L^−1^ concentration drove directional temporal shifts and increased alpha diversity, whereas the concentration of 400 μmol L^−1^showed nonspecific fluctuations.Additionally, Nitrospirota and Methylomirabilota positively correlating with POD activity and bioactive metabolites, respectively. while Verrucomicrobiota showed inverse associations. Ultimately, this work proposes a hypothesis that suitable MeJA concentration regulates host metabolism through a two-pronged associative pattern involving direct physiological elicitation and microbiome-associated shifts, offering practical insights for the microbe-assisted cultivation of medicinal plants.

## Data Availability

The datasets presented in this study are deposited in the National Science Data Bank (Science Data Bank), with the data access link: https://www.scidb.cn/anonymous/MnVNSnJl.
